# Recycling of silver nanoparticles from electronic waste via green synthesis and application of AgNPs-chitosan based nanocomposite on textile material

**DOI:** 10.1038/s41598-023-40668-7

**Published:** 2023-08-23

**Authors:** Moni Sankar Mondal, Ayon Paul, Mukitur Rhaman

**Affiliations:** Department of Textile Engineering, Khulna University of Engineering & Technology, Khulna, 9203 Bangladesh

**Keywords:** Environmental sciences, Materials science, Nanoscience and technology

## Abstract

The main thrust of this project is the fabrication of silver nanoparticles (AgNPs) from electronic waste (PCB board) and applying it on 100% cotton fabric as an antimicrobial agent. The nanoparticle formation of silver was done by green synthesis way using an aqueous leaf extract of *Eichhornia crassipes.* Furthermore, chitosan was also applied to the fabric with silver nanoparticles by coating. FTIR and SEM tests characterized the fabricated silver nanoparticles, and antimicrobial tests were followed by the disc diffusion method. The SEM analysis showed an average particle size of 76.91 nm. The FTIR analysis showed the successful reduction of silver nanoparticles and the bonding with chitosan and cellulose. Besides, the EDX reports confirmed the existence of AgNPs by indicating a strong signal in the silver region. In addition, SEM characteristics analysis confirmed the uniform deposition of silver nanoparticles. Finally, the antimicrobial property was tested against gram-positive (*Staphylococcus aureus)* and gram-negative (*Escherichia coli)* bacteria. The antimicrobial result was found satisfactory in the case of green-synthesized recycled AgNPs. However, the effectiveness was not observed to be higher than green-synthesized pure AgNPs. In this study, the zone of inhibition of AgNPs was also compared to the reference antibiotics Ciprofloxacin.

## Introduction

**Figure 1:**
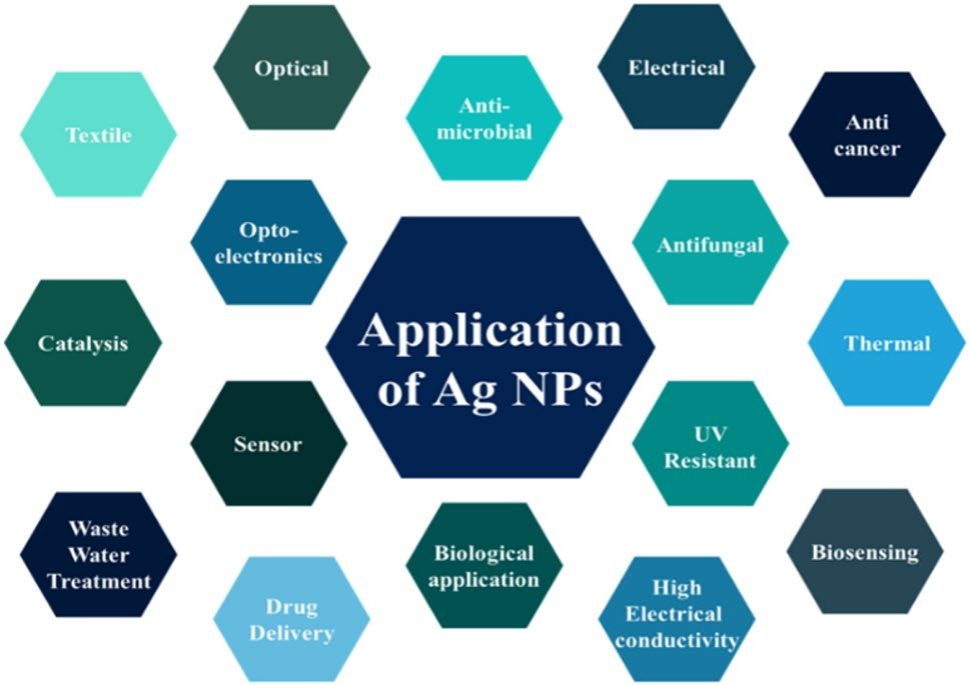
Application of AgNPs.

The demand for nanotechnology in electronics (laptops, mobile phones), textiles, medical sciences, sports, cosmetics, and construction are increasing day by day. Interestingly, it is a hot topic in the research field and fast-moving areas. The nano refers to a size of around 0.1–100 nm and has some distinctive properties that help to replace the bulk counterparts. That unique property is its tiny nanostructure containing a large surface area to volume ratio, proffering various mentionable value-added properties to any materials. Until now, nanoparticles are getting in the form of nanowires, nano-flakes, nanoparticles, nanocomposites, nanofibers, nanotubes, and nanofilms. However, among the various nanoparticles, Ag nanoparticles (AgNPs) have already gotten attention due to their interesting properties like antimicrobial, UV resistant, optical, electrical, and thermal, and high electrical conductivity^1^. Their potential applications (Fig. 1) in optoelectronics^2,3^, biomedical applications^4,5^, plasmonic^6^, sensors^7,8^, catalysis^9,10^, antimicrobial activities^11,12^, wastewater treatment^13^ and so many. However, in this research work, the main approach to analyse the antimicrobial properties of synthesized AgNPs from electronic waste**.**

Mostly, the synthesis of nanoparticles has been carried out using two approaches: (1) Top-Down and (2) Bottom-up. In top-down, the larger the particles are broken into smaller ones using grinding, ball milling, laser ablation, sputtering, etc.^14,15^. In the bottom approach, nanoparticles can be made by joining atoms, molecules, or small particles utilizing vapor phase deposition, sol–gel process, green synthesis, spinning, etc.^16,17^. However, these approaches are time-consuming, costly, and require high energy. Besides, in the bottom approach, reducing and stabilizing agents are used to form AgNPs. The most common reducing and stabilizing agents are sodium borohydride (NaBH_4_), ascorbate, N, N-dimethylformamide (DMF), poly(vinylpyrrolidone), poly (vinyl alcohol), poly (ethylene glycol), etc.^18,19^ respectively. This chemical can form a toxic byproduct during the synthesis process, resulting in environmental pollution, which is unwanted. As a result, the green synthesis process has come to the screen providing many advantages like requiring less energy and no toxic chemical. Besides, it is a cost-effective and fast synthesis approach. Green synthesis of silver nanoparticles can be obtained by using plant extract, microorganisms like bacteria^20,21^, fungi^22,23^, and yeast^24–26^_._ Recently an approach has been executed to fabricate AgNPs through green synthesis using Natural Gums such as *Mimosa pudica, Tragacanth, Acacia, Cashew, Ghatti, and Olibanum.* Their synthesized AgNPs showed higher antimicrobial properties as well as higher sensitivity and colorimetric detection of mercury ions in water samples^27^*.*At present, the synthesis of silver nanoparticles by plant extract has been proliferating because this is an eco-friendly, cost-effective, and time shortage process. Different plant parts are used to synthesize silver nanoparticles, such as roots, leaves, flowers, seeds, etc^28–30^. The main reasons for using various plant parts are their wide range of accessibility, safety, and the presence of enormous active compounds such as proteins, amino acids, enzymes, polysaccharides, alkaloids, tannins, polyphenols, saponins, terpenoids, and vitamins that contribute to the reduction of silver ions^31–33^. Due to the presence of plenty of organic molecules, no extra reducing or stabilizing agents are used in this process. Synthesis of silver nanoparticles from plant extract has been performed in two stages: plant extract preparation and silver nanoparticle synthesis via prepared extract solution. In addition, with the recent development of nanoparticles from the perspective of functional activities, multifunctional nanoparticles are far ahead of conventional nanoparticles. The multifunctionality of nanoparticles refers to their ability to execute several functional synergistically^34^. In the case of silver nanoparticles derived through green synthesis, the properties like plasmonic and antibacterial effects was found by surface modification with diverse surfactants to make it multifunctional^35^. This development contains the immense possibility for therapeutic application, such as cancer treatment, diagnostics, and neuropathologies, inhibiting HepG2 cell proliferation^36^.

A good number of researches have been done on AgNPs’ antimicrobial properties where Ag was turned into its nanoform through green synthesis. For example, animal blood serum^37^, soluble soybean polysaccharide (SSPS)^38^. *Achillea millefolium* leaf extracts^39^, black tea^40^, *Conocarpus Lancifolius* plant extract^41^ have been applied to synthesize silver NPs with highly antimicrobial and antiviral properties.Besides, some researchers also employed silver nanoparticles with cellulose-based products to impart antibacterial properties. Besides, some researchers also employed silver nanoparticles with cellulose-based products to impart antibacterial properties. In this way, *Moringa oleifera* leaf-extract, -mediated bio-fabricated silver NPs enhanced the antibacterial properties of cellulose-based fabrics ^42^, whereas AgNPs, synthesized through a chemical reduction, incorporated into wipes that showed efficiency in combating coronaviruses^43^. Another approach, silver nanoparticles impregnated polysaccharides substrates (cellulose powder (CP), microcrystalline cellulose (MCC), carboxymethyl cellulose (CMC) and chitosan (Chit)) were developed for the evaluation of antibacterial activity against Gram-negative *Escherichia coli* (E. coli) and Gram-positive *Staphylococcus aureus* (S. aureus) bacteria and satisfactory result was obtained from this work^44^. Another study showed that electrospinning method was used in the sysnthesis of silver nanparticles, embedded in polystyrene nanofiber to utilize as wound healing biomaterial and antibacterial. This fabricated nanocomposite AgNPs-PS had shown better antibacterial activity with the increased AgNPs amount^45^.The synthesis of AgNPs was successfully carried out using the Turkevich method, and chemical reduction method from waste-printed circuit boards and electronic scrap, respectively^46,47^. But all of them focused on the synthesis of AgNPs only. However, no significant research work has been reported on the synthesis of AgNPs from electronic waste using the green synthesis method, as well as analysis of its antimicrobial activity on textile materials. This study involves recycling AgNO3 from PCB boards (electronic waste) to synthesize AgNPs following the green synthesis method where water hyacinth plant extract was used and applying these AgNPs on fabric as coating form with chitosan to increase the crosslinking and antimicrobial efficacy. To our knowledge, this approach is still relatively new among the researchers. One the other hand, Water hyacinth, a free-floating aquatic plant, is available in Bangladesh. It belongs to the Pontederiaceae family and is native to South America^48^. It grows rapidly, creates troubles for water transportation, and lessens oxygen levels in aquatic environments^49,50^. However, this plant has medicinal benefits. This plant can be used for anti-inflammatory, antifungal, and antibacterial purposes. Additionally, it can be applied as a hair fragrance and for treating cholera, sore throat, and snake bites^51^. This situation motivates the authors to utilize the water hyacinth beneficially. In the case of electronic waste, A 2018 survey shows 533 tons of silver were used in electronic industries worldwide^52^. According to e-waste generation studies, the quantity of e-waste created globally in 2018 was around 53.6 million metric tons^53^. By 2021, the volume of e-waste was predicted to increase at an annual rate of 3 to 4%^54,55^. According to Statista, the electronic waste generation in 2022 was 59.4 million metric tons^56^. Still, the total quantity of e-waste collected for recycling was just 11.70%. The fast growth of electrical and electronic equipment (EEE) functions, appealing consumer designs, marketing, and compatibility has increased the number of environmental and industrial issues, such as pollution caused by poor landfilling and incineration procedures. Various electronic products, including refrigerators, laptops, mobile phones, televisions, and others, utilize printed circuit boards (PCBs) to execute their functionalities. These boards comprise various materials, such as silver, copper, zinc, iron, and others. However, along with these materials, heavy metals and halogenated materials are also present in PCBs. The usage of PCBs accounts for approximately 3% of the total e-waste generated. Improper disposal of these materials can create adverse effects on the environment. Therefore, proper handling and disposal of PCBs are necessary to prevent environmental damage^57^.Overall, this work is intended to highlight the achievement of synthesizing AgNPs using both green and chemical synthesis methods. In our process, AgNO_3_ solution, the precursor of AgNPs, was prepared by dissolving Ag in the leaching agent nitric acid, and finally the Ag was collected from e-waste, creating a more environmentally friendly and sustainable method for synthesizing these nanoparticles. Moreover, the antimicrobial properties of the recycled AgNPs-coated fabric were compared with pure AgNPs coated fabrics . Additionally, these green-synthesised AgNPs can be implemented as an alternative source of antimicrobial agents for the textile industry. However, the synthesis of pure AgNPs from electronic waste was quite challenging in our research work due to the presence of other materials. The presence of foreign materials created difficulties during the synthesis of nanoparticles, sample preparation, and characterization of the resultant nanoparticles.

## Result and discussion

### UV–Vis spectra analysis

**Figure 2:**
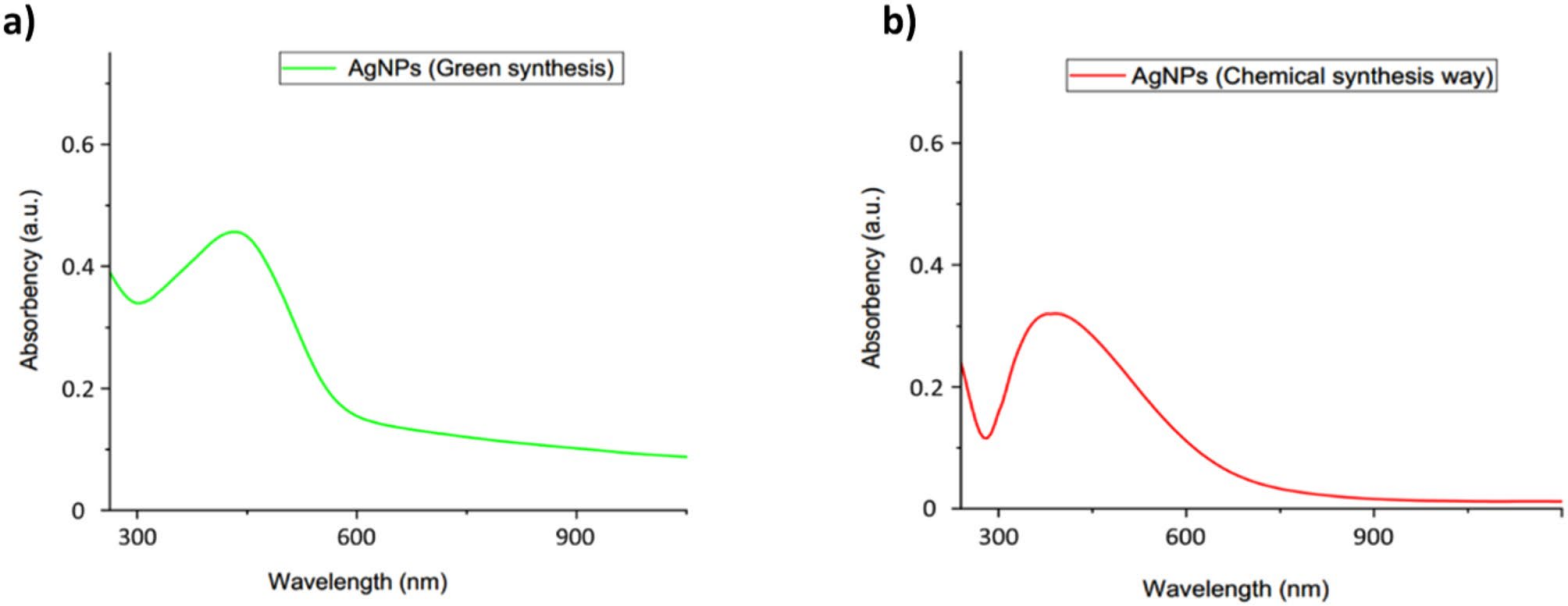
UV–Vis of (**a**) AgNPs green synthesis (**b**) AgNPs Chemical synthesis.

The UV–Vis test was used to identify the formation of AgNPs. This testing method seems to be very fast, handy, and sensitive to detect nanoparticle formation^58^. During the green synthesis process, the color change from pale yellow to dark brown is attributed to the nanoparticles' formation of silver. Based on UV–Vis spectra, the maximum absorbance was found at 445 nm for the green synthesis process (Fig. 2a) and 425 nm for chemical synthesis (Fig. 2b). AgNPs are known to exhibit a UV–Vis absorption maximum in the range of 400–500 nm^59^. So, the result clearly stated that the nanoparticle formation occurred throughout the process. The green synthesis process shows much higher absorbency than the chemical synthesis process. Due to plants' different phytochemicals, phenols, proteins, alkaloids, amino acids, carbohydrates, flavonoids, glycosides, tannins, and terpenoids causes higher absorbance. Furthermore, the UV–visible spectrophotometer test displayed a single peak that denoted silver nanoparticles' spherical shape corresponding to the Mie theory^60^.

### SEM and EDX analysis

**Figure 3:**
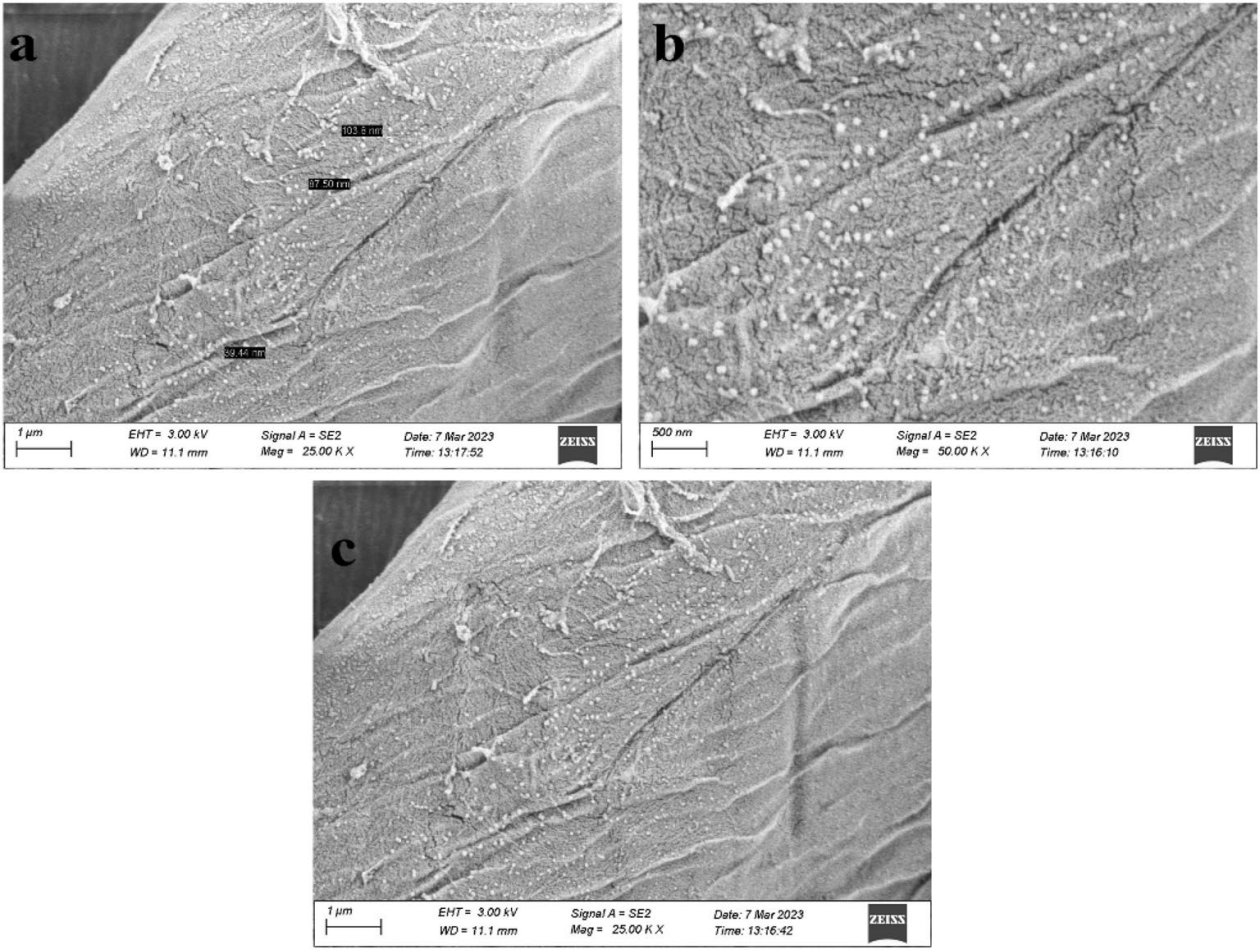
(**a**) Indicate nanoparticle size of Ag through SEM, (**b**) Surface morphology observation through SEM (Green Synthesis), (**c**) Surface morphology observation through SEM (Chemical Synthesis).

The coated fabric surface morphology was studied by doing SEM test. The nanoparticle size and coating uniformity pictures were taken at different magnifications. Most of the shapes of the particles were found to be round and spherical. Figure 3a shows a size range from 39.44 nm to 103.8 nm. On average, the particle size is 76.91 nm. From this outcome, we can say the synthesis process of AgNPs was valid and showed a reasonable result.

In Fig. 3b and c**,** It can be observed that these silver nanoparticles particles are acutely spread over the surface without agglomeration. As a result, the nanoparticles filled the voids and spaces between the cotton fibers, resulting in evenly distributed on the surface for both green and chemical synthesis.

**Figure 4:**
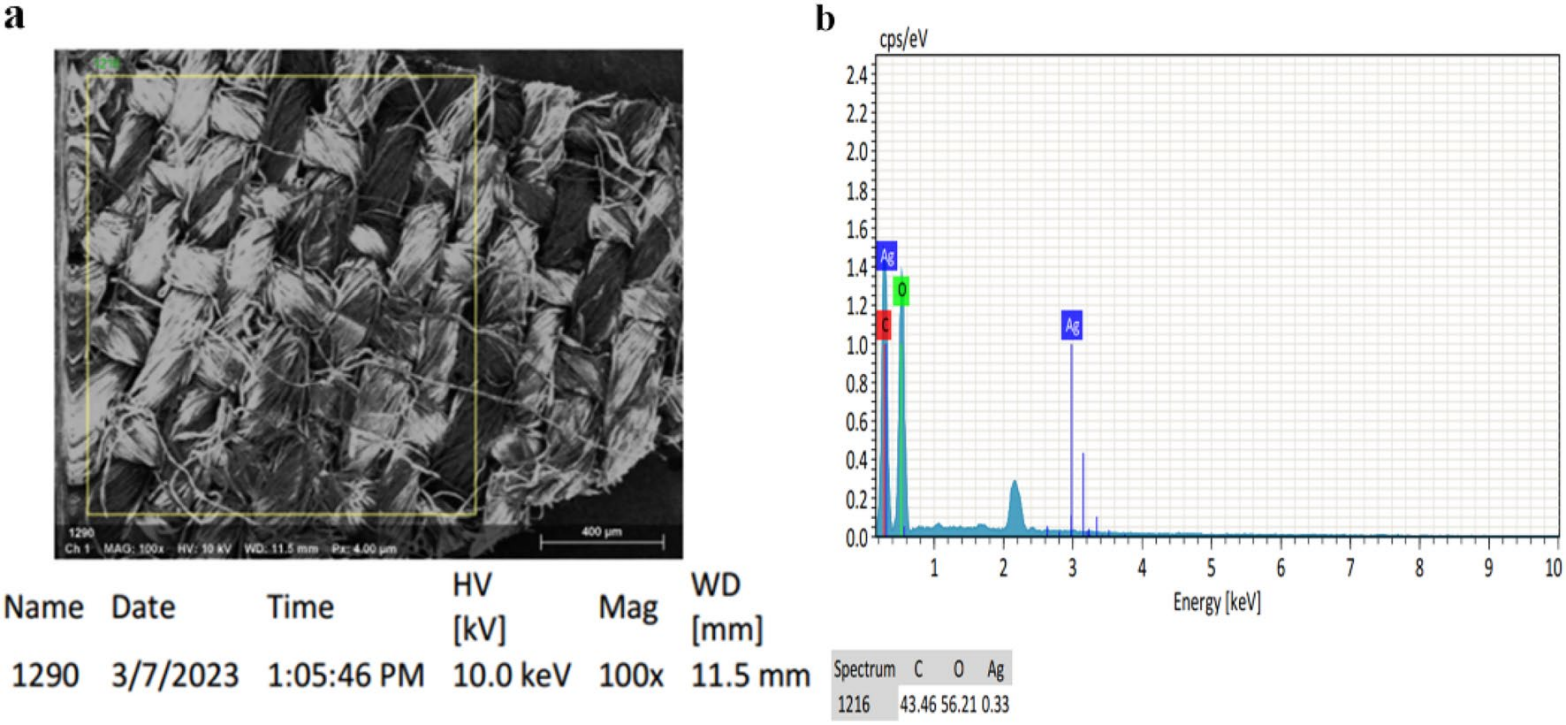
EDX spectrum of AgNPs from extract *Eichhornia crassipes* (**a**) AgNPs-chitosan coated fabric surface, (**b**) Indicating spectrum of Ag.

Figure 4a and b illustrates the sample's elemental composition, which confirmed the formation of AgNPs by showing a strong signal in the silver region. The optical absorption peak of AgNPs generally shows approximately 2.99 keV to 3 KeV^61,62^. Here the peak of AgNPs was found at 2.99 keV. Besides Ag, carbon, and oxygen materials, spectra were also detected due to the extracellular organic materials from the plant extract.

### XRD analysis

**Figure 5:**
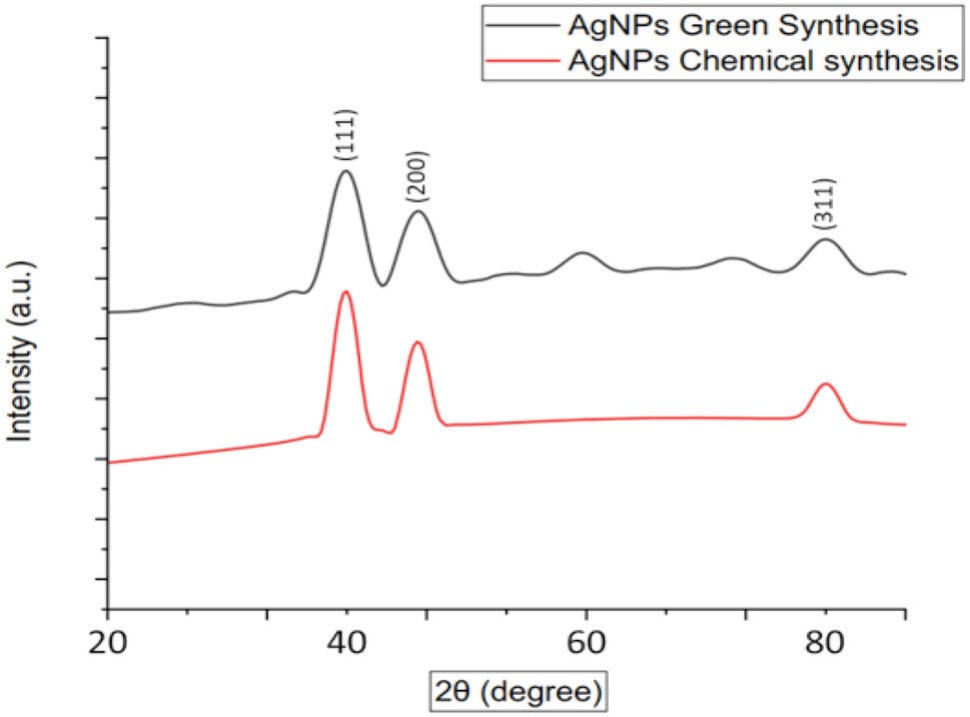
XRD patterns of AgNPs.

From this XRD pattern as shown in Fig. 5, the crystalline form of the AgNPs was studied. For AgNPs Green synthesis several peaks are found at 2θ degree respectively 38°, 46°, and 78° which corresponding to (111), (200), (311). Similarly, for AgNPs chemical synthesis peaks were identified at 2θ degree 38°, 46°, and 78° which is corresponding to (111), (200), (311). The results of our study are in line with other published literature; the crystal nature of silver nanoparticles synthesized with *Tagetes erecta*^63^, *Urtica dioica*^64^, *Aegle marmelos*^65^, *Carduus crispus*^66^, *Serratia nematodiphila*^67^ was face-centered cubic with diffraction peaks of (111), (200), (311) respectively. Some weak peaks are observed in green synthesized AgNPs, which could be some organic residual remaining from organic or inorganic parts of the precursors^68,69^. The average crystallite size was calculated using Scherrer's equation. The average crystallite size of AgNPs was found to be around 46–49 nm of both origins. Furthermore, this finding is consistent with the observed sizes in FE-SEM analysis.

### FTIR analysis

**Figure 6:**
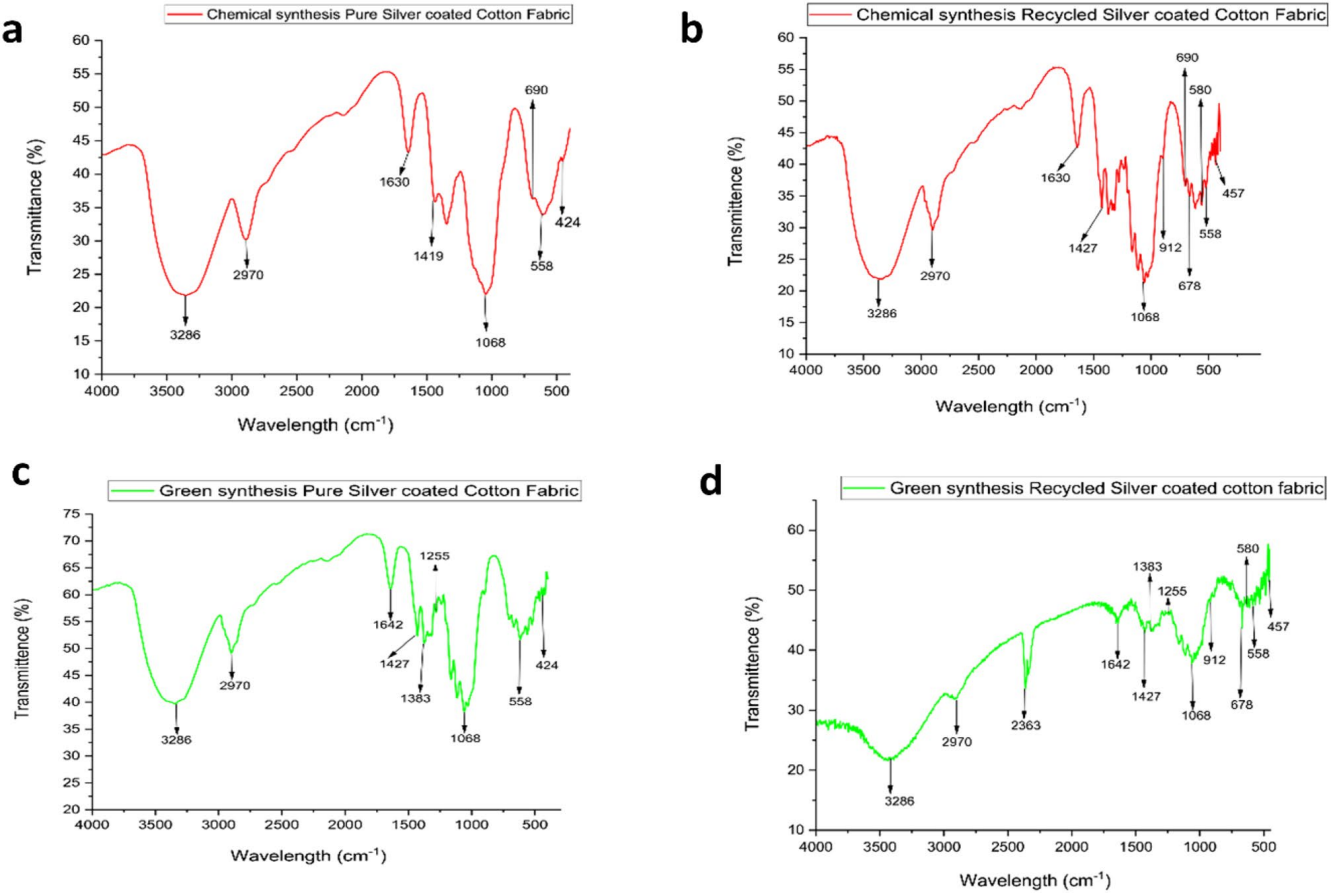
FTIR report of (**a**) Chemical synthesis of Pure Silver nanoparticle coated Cotton Fabric, (**b**) Chemical Synthesis of Recycled Silver nanoparticle coated Cotton Fabric, (**c**) Pure AgNPs coated cotton fabric in green synthesis, (**d**) Recycled AgNPs coated cotton fabric in green synthesis.

FTIR analysis of pure silver coated cotton fabric was carried out to identify the functional groups present and is shown in Fig. 6a**. **FTIR spectrum containing AgNO_3_ shows peak shifts with intense absorption bands at 424, 558, 690, 1419, 1642, 2970, and 3286 cm^−1^. The presence of a peak at 3286 cm^−1^ may be attributed to the O–H stretching vibrations stretching, indicating a hydroxyl group’s existence. The band detected at 2970 cm^−1^ corresponds to the C–H stretching (sp^3^ carbon) of cellulose^70^. The peaks found on 1642 cm^−1^ and 1419 cm^−1^ indicate the presence of amine group and aliphatic group, respectively^71^. Another peak detected at 1068 cm^−1^ are assigned to the C–O stretching of the alcohol group^72^. 690 cm^−1^ corresponds to the halo compound due to the presence of Sodium borohydride during chemical synthesis. Also, a peak at 558 cm^−1^ indicates the (C–C) alkane stretching. Finally, the peaks observed at 424 cm^−1^ attribute to the functional groups that are responsible for the synthesis of cellulose-capped Ag-NPs (C–Ag–NPs). Additionaly, the peaks detected at 3286 cm^−1^ and 1642 cm^−1^ confirm the interaction takes place through the hydroxyl group (–OH) and the amine group (–NH) of the chitosan polymer chain^73^.

The results of FTIR analysis **(**Fig. 6b**)** showed the presence of some common peaks in recycled Silver coated cotton fabric at 3286 cm^−1^, 2970 cm^−1^ and 1642 cm^−1^ as pure silver coated cotton fabric which corresponds to the stretching vibrations of hydroxyl, cellulose and amine groups, respectively. One the other hand, some new peaks were also observed in this FTIR test. The peaks 1427 cm^−1^ indicate C=C aromatic stretching vibration^74^, Whereas C=C bending out of-plane is present in AgNPs was observed from the band at 912 cm^−1^^75^. The results of FTIR analysis showed the presence of several foreign materials Cu, Zn, and Fe were confirmed from the bands at 678, 580, 457 cm^−1^^76–78^.

Bio-synthesised pure AgNPs coated cotton fabric **(**Fig. 6c**)** shows different peaks under the FTIR test. The peak found at 3286 cm^−1^ are assigned to the O–H stretching vibrations. The C-H stretching (SP^3^ carbon) of cellulose was detected in 2970 cm^−1^ wavelengths. 1642 cm^−1^ and 3286 cm^−1^ present the interaction between AgNPs and chitosan, which takes place through the hydroxyl group (–OH) and the amine group (–NH) of the chitosan polymer chain. Besides the 1427 cm^−1^, 1383 cm^−1^, 1255 cm^−1^, and 1068 cm^−1^, respectively attributed to the phenolic group, N–O stretch of strong aliphatic amines, C–O of alcohol groups, and stretch of the alcohol group. So, it can be inferred that the major compounds detected in AgNPs coated fabric are phenolic, aliphatic amines, and alcoholic groups corresponds to the reducing and stabilizing groups of *Eichhornia crassipes*. Finally, the bond between cellulose and AgNPs was ensured by observing the peak 424 cm^−1^ (cellulose–capped Ag-NPs(C-AgNPs)).

Like the chemical synthesis of recycled silver chitosan-coated cotton fabric, the green synthesis method presents **(**Fig. 6d**)** the similar FTIR bands for foreign metals. These metals peaks were observed at 2363, 678, 580, and 457 cm^−1^, respectively, attributed to C=N, Fe–O, and Zn–O. Similarly, the peaks found on 1642 cm^−1^ and 3286 cm^−1^ shows the interaction between AgNPs and chitosan. The result of FTIR confirmed the presence of various reducing and stabilizing agents by observing the peaks at 1427 cm^−1^ (phenolic group), 1383 cm^−1^ (N–O stretch of strong aliphatic amines), and 1255 cm^−1^ (C–O of alcohol groups). 912 cm^−1^ and 558 cm^−1^ are attributed to the C=C out of-plane bending and C–C alkaline stretching were found in the treated sample.

The chemical and molecular similarities between cellulose and chitosan lead to their strong attraction. The hydroxyl group (–OH) present in cellulose and the amine group (-NH) found in the chitosan polymer chain establish hydrogen bonding between the two polymers. These intermolecular interactions between cellulose and chitosan primarily rely on hydrogen bonds and Van der Waals forces. However, under specific conditions and cellulose treatments, there is a possibility for the formation of ionic and/or covalent bonds^79^.

The presence of Ag + metal ions facilitates the bonding between the hydroxyl (–OH) groups of cellulose and the metal cations. In this process, water molecules are involved in the coordination sphere of the metal ions while simultaneously forming hydrogen bonds with the -OH groups of cellulose^80^.

### Bio reaction mechanism

**Figure 7:**
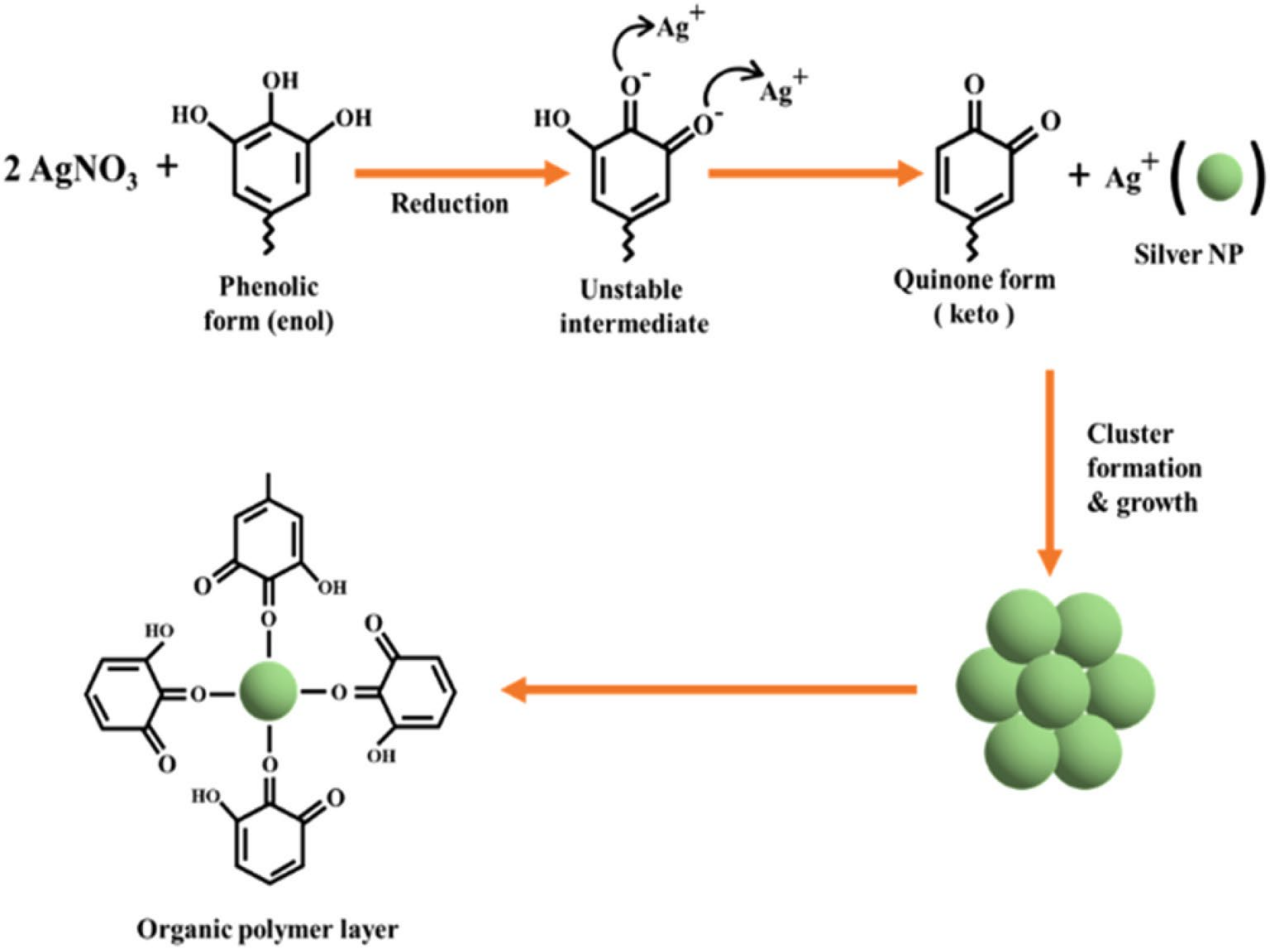
Mechanism of green synthesized silver nanoparticles.

The water hyacinth contains several phytochemicals, including phenols, protein, alkaloids, amino acids, carbohydrates, flavonoids, glycosides, tannins, and terpenoids^81^. These phytochemicals are responsible for reducing and stabilizing silver ions into AgNPs. Many scientists stated that different biomolecules are accountable for the reduction and stabilization process of AgNPs. According to Aymn Yaseen Sharaf Zeebaree et al., the above-mentioned phytochemicals contain many hydroxyl (OH) groups in their molecular structure, facilitating the redox process. These phytochemicals contribute to metal reduction by donating an efficient number of electrons^82^. Another study showed that the water hyacinth contains many phenolics and flavonoids and the hydroxyl groups of flavonoids (quercetin) facilitate silver ions into nano size by reducing. Quercetin exhibits keto-enol isomerism and the hydrogen atom released during this conversion of enol to keto forms in quercetin helps convert the ion into metallic silver nanosized and stabilizes the AgNPs^83^. The complete understanding of the formation process of silver nanoparticles (AgNPs) from plant extracts is yet to be fully clarified due to the presence of multiple functional groups. However, three stages of plausible mechanism for the synthesis of AgNPs can be delineated in Fig. 7**:** (1) the conversion of silver ions into silver atoms through reduction, (2) the initiation of nucleation and subsequent enlargement of silver atoms to generate nanoparticles with distinct size and morphology, and (3) the ultimate safeguarding of the formed AgNPs to prevent their aggregation^84^.

### Antimicrobial analysis

**Figure 8:**
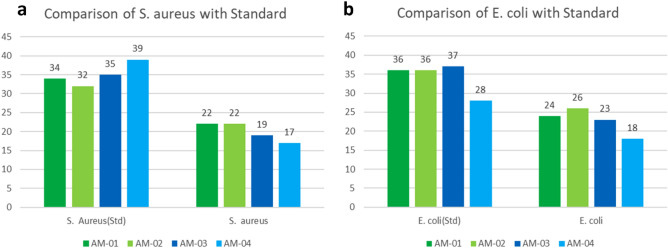
Comparison of Antimicrobial efficacy of treated samples with standard against (**a**) *S. aureus,* and (**b**) *E. coli.*

In the investigation of antibacterial activity, two bacteria, namely Staphylococcus aureus and Escherichia coli, were used by employing the disc diffusion method. Table 1 and Fig. 8a and b show the efficacy of AgNPs-chitosan-coated fabrics against *E. coli* and *S. aureus*. The film discs demonstrated the reduction of bacterial growth known as inhibition zones. In this result, the AM-01, AM-02, AM-03, and AM-04 are the code names of Recycled AgNPs in Green Synthesis, pure AgNPs in Green Synthesis, Recycled AgNPs in chemical Synthesis, and Pure AgNPs in chemical synthesis.

**Table 1:** Result of Antimicrobial.

Sample name	Standard Ciprofloxacin (10 µg/disc)		Control		Sample	
	*S. aureus*	*E. coli*	*S. aureus*	*E. coli*	*S. aureus*	*E. coli*
AM-01	34	36	0	0	22	24
AM-02	32	36	0	0	24	26
AM-03	35	37	0	0	19	23
AM-04	39	28	0	0	17	18

**Figure 9:**
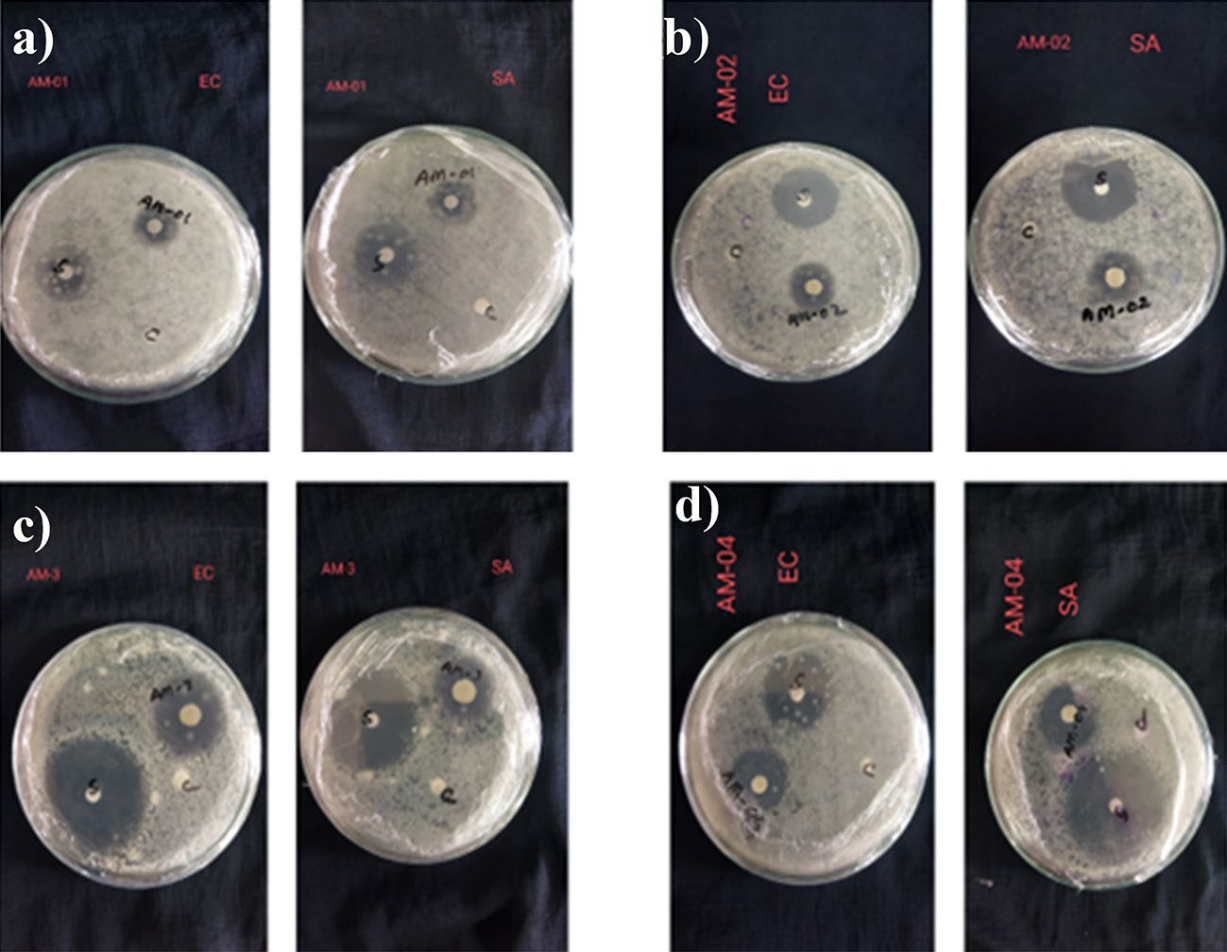
Inhibition zone of **(a)** recycled AgNPs in green synthesis, **(b)** pure AgNPs in green synthesis, **(c)** recycled AgNPs in chemical synthesis, and **(d)** pure AgNPs in chemical synthesis fabrics sample against *S. aureus* (SA) *and E. coli* (EC).

Here the control sample marked as C in Fig. 9a–d represents the negative control as well as sterilization of that agar media on the petri plate. Ethanol was used as a solvent to sterilize the Petri dish so that a better result could be obtained. Also, Ethanol has its own antimicrobial effect. Finally, heat was applied on the petri plate. As a result, the ethanol was vaporized and the media was sterilized.

The Ciprofloxacin (10 µg/disc) was used as a positive control marked as **S** in Fig. 9a–d to compare the inhibition zone with the sample. The antibiotic, Ciprofloxacin, creates a standard inhibition zone. A decision can be made on antimicrobial results by comparing the inhibition zone, Silver nanoparticles inhibited *E. coli* more than *S. aureus,* which is depicted in Fig. 8**,** and the values of the inhibition zone are shown in Table 1. This is due to the characteristics of *E. coli,* which is a gram-negative bacterium with a thin peptide glycan layer and a more negative charge. This situation makes it easier for AgNPs to penetrate the layer and destroy it^85^.

In both cases, the green synthesis of pure silver nanoparticles **(**Fig. 9b**)** shows the best antimicrobial effect. Here, the green synthesis process leaf extract provided stabilizer and reducing agents such as phenolic compounds, alkaloids, carboxylic groups, and flavonoids^86^. These compounds also have an antimicrobial effect. So, AgNPs' antimicrobial effect with these compounds creates a reasonable outcome than the chemical synthesis of pure silver nanoparticles (Fig. 9d**)**.

However, the AM-01, Recycled AgNPs in Green Synthesis (Fig. 9a) contain some metals (Zn, Fe, Cu), which were identified in FTIR and also showed the antimicrobial effect. So, the synergistic effect of all metals with AgNPs should be shown as the best antimicrobial effect. As a matter of fact, while conducting the green synthesis of recycled silver, the P^H^ was not appropriately controlled. The media was excessively acidic. As a result, the slow nucleation rate results in a larger particle size. Thereby less antimicrobial effect^87,88^. In fact, the green synthesis of recycled AgNPs demonstrated a greater antimicrobial effect compared to the chemical synthesis of recycled AgNPs (Fig. 9c).

### Effect of AgNPs treatment on tensile strength

**Figure 10:**
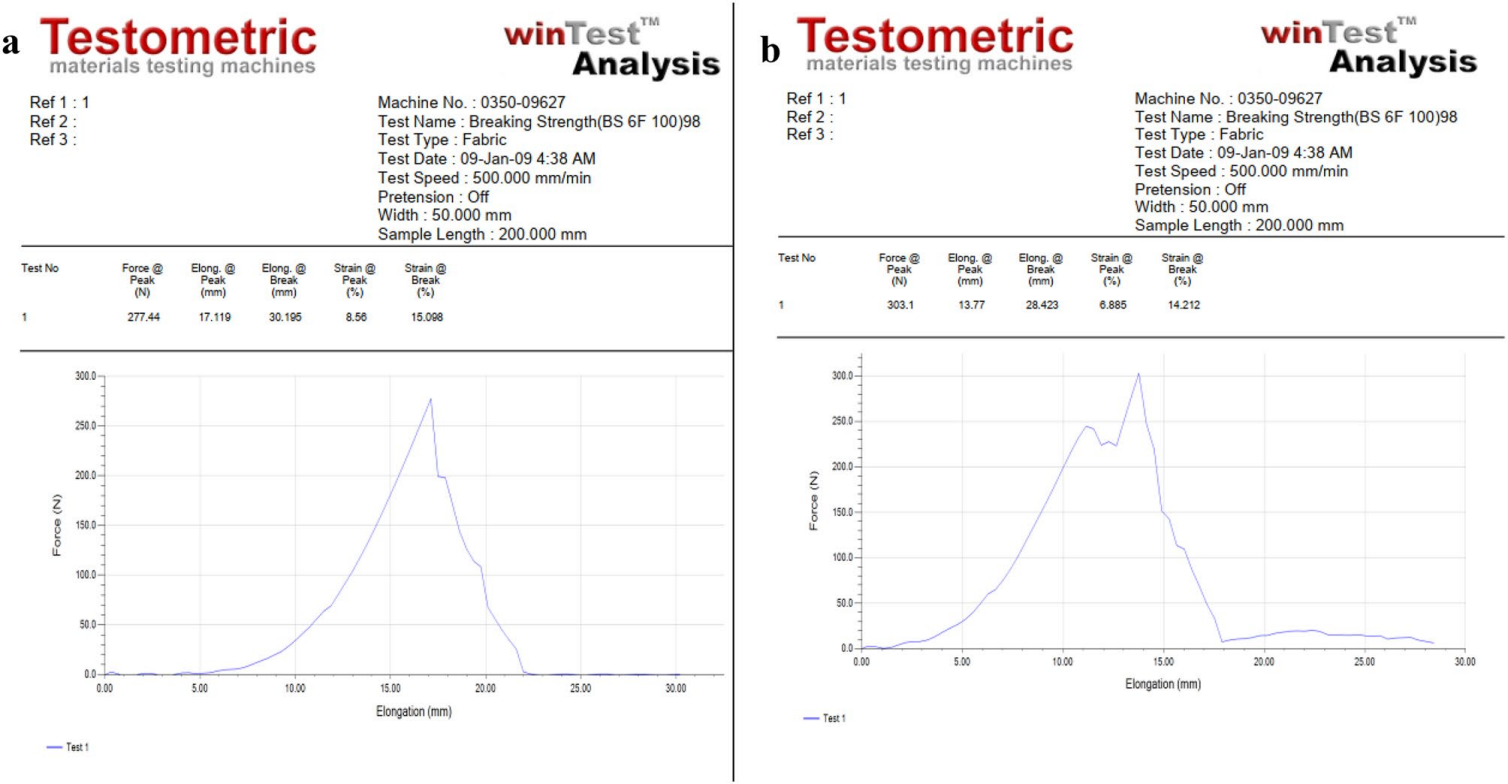
(**a**) Tensile strength of the untreated fabric, (**b**) Tensile strength of the treated fabric.

The effect of AgNPs on cotton fabric physical properties, respectively, Tensile strength and crease recovery, have been investigated. The findings present that integrating AgNPs into the fiber's structure increases the fabric's load-bearing capacity. The reason can be described as the ability of the tiny nano silver particle to infiltrate the polymer molecules, thereby acting as fillers or crosslinking agents. The filler or crosslinking types of activities help load sharing when pressure is applied. As a result, a better tensile strength was found for treated fabric (Fig. 10b) than untreated fabric (Fig. 10a).

### Effect of AgNPs Treatment on crease recovery, fabric GSM and thickness

This integration of AgNPs made the fabric more rigid and enhanced the crease recovery angle by around 37%, which is presented in Table 2. The overall result showed a slight improvement in crease recovery angle.

**Table 2:** The crease recovery angle in weft way.

Untreated	Mean	Treated	Mean
80°	75°	100°	102.5°
70°		105°	

The treated and untreated samples showed a minimal difference in GSM and thickness values. As the fabric was coated with AgNPs and chitosan, its weight and thickness increased, thereby increasing its overall density. The untreated and treated fabrics GSM and thickness are tabulated in Table 3.

**Table 3:** GSM and thickness value of untreated and treated fabric.

Untreated	Treated
GSM	
96.4 gm	97.9 gm
Thickness	
0.27 mm	0.29 mm

## Methodology

### Materials

The 100% cotton woven pretreated fabric was used, which GSM was 96.4, comprising the 40-count yarn in both the warp and weft direction. The EPI and PPI of the fabric were respectively 120 and 70. The construction was analyzed and found to be a 1/1 plain weave design. As an electronic waste, PCB boards were used. Silver nitrate, Ascorbic acid, and Nitric acid were purchased from Sigma Aldrich. Sodium Hydroxide and Sodium Borohydride were collected from authorized distributors of Merck, Bangladesh.

### Collection and studies on plant material

**Figure 11:**
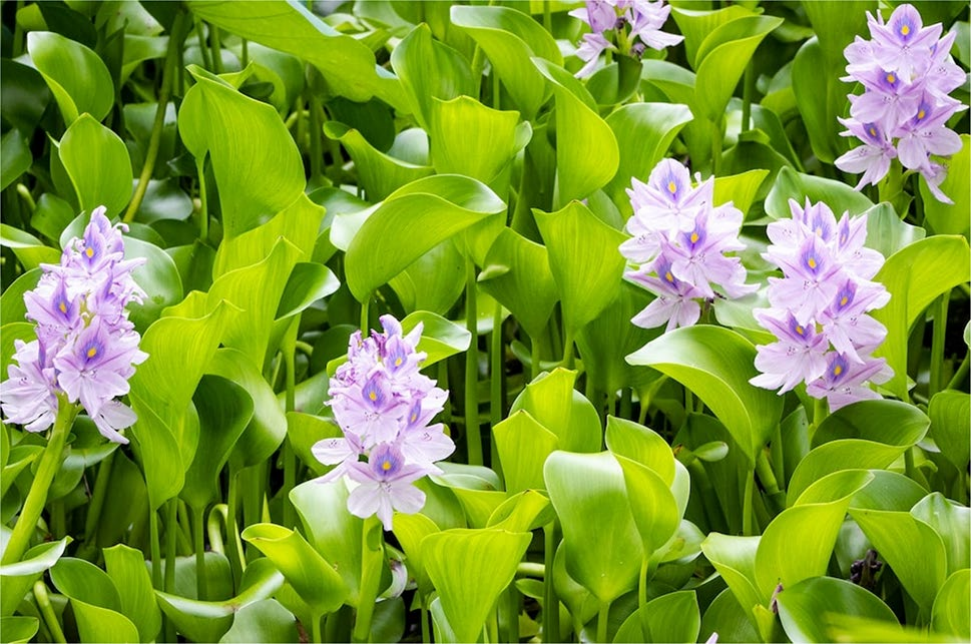
Water hyacinth plant.

The collection of *Eichhornia crassipes* (plant) material was performed according to institutional, national, and international guidelines. Plant studies and all experimental procedures were performed in conformity with applicable institutional, national, and international guidelines. Plants (Herbarium number: 66769—*Eichhornia crassipes((Mart.)solms*. as shown in Fig. 11) were identified by Md. Hashem, Associate Professor, Department of Botany, Khulna Govt. Girls College, National University, Bangladesh.

Water hyacinth (*Eichhornia crassipes*) leaves were obtained from a pond of Khulna University of Engineering & Technology, Khulna, Bangladesh.

No certification or permission was needed to collect the samples because the questioned species is extensively spread throughout the nation; nonetheless, the Khulna City Corporation and University Administration Authority were consulted before sampling.

## Method

### Preparation of AgNO_3_ from electronic waste

Electronic waste, including motherboards, circuit boards, and PCB boards, was collected from an electronic shop located in Khulna, Bangladesh. The silver particles were extracted from this waste and weighed at 3.6 g using an electronic balance. The silver particles were then added to a solution of 0.5 M 70 ml HNO_3_ acid and left to dissolve for three days to ensure complete dissolution of the silver particles. The resulting mixture was filtered and stored for further processing.

### Preparation of pure AgNO_3_ solution

For pure AgNO_3_ solution preparation, a 0.2 M AgNO_3_ solution, 0.85 g of AgNO_3_ powder, was taken and dissolved in 25 ml of deionized water.

### Preparation of plant extract and green synthesis of silver nanoparticles

Water hyacinth leaves were thoroughly washed with tap water to remove any dust and soil particles and were subsequently air-dried under sunlight for 3 days. Once dried, the leaves were ground into a fine powder, weighed at 3.036 g, mixed with 350 ml deionized water, and heated at 80 °C while continuously stirring at 250 rpm for one hour. The resulting mixture was then filtered through filter paper, and the extract was stored at ambient temperature for further processing. This extraction process was conducted in accordance with standardized procedures to ensure the purity and efficacy of the resulting extract.

The prepared plant extraction was used to reduce Ag ions from the AgNO_3_. To synthesize AgNPs, 30 mL of plant extract was treated with 30 mL of AgNO3 (0.2 M) solution and allowed to react at room temperature for 1 h in direct sunlight. During this, the color of the reaction mixture was changed to brown, a color as an indication of the synthesis of AgNPs^83,89–92^.

### Chemical synthesis of AgNPs from electric waste and pure AgNO_3_ solution

A 0.2 M solution of silver nitrate (AgNO3) was loaded into a burette to synthesize Silver NP chemically. In contrast, a conical flask containing a 0.2 M solution of sodium borohydride (NaBH_4_) was prepared with an ice cube to lower the reaction rate. The AgNO_3_ solution was then slowly added dropwise into the NaBH_4_ solution. To stabilize the synthesized Ag NPs, 1% ascorbic acid was subsequently introduced into the solution^93^.

### Preparation of chitosan

The shrimp shells were collected from the local market for Chitosan preparation, washed, and dried in sunlight. To remove the protein from the shells, 14.6 g of shrimp shell was treated with 25% NaOH solution for 1 h at 60 °C, then washed with water and dried. The resulting deproteinized chitin shell was then treated with 10% HCl solution for 2 h at 60 °C, then washed with water and dried. The chitin was then converted to chitosan by treating it with 25% NaOH solution for 1 h at 120 °C and 350 rpm, followed by washing with water and drying^94^.

### Fabric pretreatment

For pretreatment of 100% cotton woven fabric, 2 g/L NaOH (2% stock solution), 4 g/L H_2_O_2_ (2% stock solution), 1 g/L Detergent (1% stock solution), 1 g/L Sequestering agent (1% stock solution), 1 g/L Stabilizer (1% stock solution) was used. The scouring bleaching process was done at 100 °C for 40 min^95^_._

### Sample preparation

A 0.5% (wt%) chitosan solution was prepared in deionized water with a 5% acetic acid solution. The fabric sample was then immersed in the chitosan solution for 48 h at room temperature. After that, padding was carried out using a padding mangle, and the sample was cured at 120 °C for 4 min. Next, the cured sample was immersed in a filtered AgNPs solution at room temperature for 3 days. Finally, the sample was padded again and cured at 120 °C for 4 min.

### Characterization

The synthesized AgNPs through green synthesis using Water Hyacinth. The formation of AgNPs was identified by observing a color change from light yellow to dark brown. The stability of the AgNPs synthesis process was examined through UV–Vis spectra using a Cole Parmer UV/Visible spectrophotometer (Model No. Unico – Mfr # CP-IS28E, USA), having a resolution of 1 nm with a range of 300 to 1000 nm. Fourier Transform Infrared Spectroscopy (FTIR Analysis) was used for identifying various organic, polymeric, and inorganic materials by following ASTM- E168 standard. For each spectrum, the transmittance was recorded from 400 to 4000 cm^−1^ by accumulating full scans with a resolution of 70 cm^−1^. The scanning electron microscope test was conducted on Zeiss Sigma 300 SEM, manufactured by Zeiss (Germany), with the microanalysis system EDX model to identify the elemental composition. Microscope resolution was 1.2 nm at 15 kV (magnification of 10X to 1,000,000X), with a depth resolution of 1 µm. X-ray diffraction patterns for AgNPs (without sonication) samples were performed on a diffractometer Rigaku (Smart lab, Japan) with Cu Kα radiation (λ = 0.154 nm) at 40 kV and 30 mA to understand the structural phase of AgNP. XRD data were collected from 2θ = 10°–90° at a scan rate of (0.02°).

From XRD data, the crystallite diameter (Dc) of Ag nanoparticles was calculated by using the Scherrer equation (Dc = Kλ/βcosθ), Where K is the dimensionless shape factor for spherical crystallite with a value of 0.94, β is the line broadening at FWHM (full-width half maximum) of maximum intensity in radians, θ is the Bragg angle at peak position, and λ (X-ray wavelength) is constant radiation with the value of 0.15406 nm.

### Antimicrobial

The antibacterial activity of silver-chitosan-coated fabric was determined by following the disc diffusion method^39^. Gram-positive (*S. aureus*) and gram-negative (*E. coli*) bacteria were used to conduct this test. The whole testing procedure was done in the Laboratory of Pharmacy Department of Khulna University by their lab operators. At first, the nutrient agar medium was prepared and kept on the autoclave at 121 °C. Here they emphasize the higher importance of sterilization of every material used during the test. A sterilized loop was used to collect a small number of bacteria from the fresh culture and transfer it into distilled water. This process is called inoculation. From this preparation, a certain amount of dilution was transferred to a petri dish containing a solid nutrient agar medium. Then, mixing was done for the proper and uniform distribution of the bacteria. Next, the Petri plate was kept at 37 °C for 24 h to cool and solidify the agar medium. Due to these steps, a suitable surface was created for bacterial growth. After that, the sample was cut and placed on the Petri plate. In this method, Standard Ciprofloxacin (10 µg/disc) was used as an antibiotic placed on that Petri plate, and the plate was incubated at 35 °C for 24 h. Finally, the zone of inhibition around the antibiotic and sample was observed and measured.

### Fabric thickness test

An advanced thickness gauge (meter) was used to measure the thickness of the sample fabrics. The testing was done in accordance with the ASTM-D1777 test standard.

### Evaluation of tensile strength

This test is done in the Fabric Lab, Department of Textile Engineering, KUET, ASTM-D 5034 standard. The machine model is Testometric. Specimen Size: 200 mm X 50 mm. Generally, in UTM machine contains two jaws, upper and lower. The sample was clamped on between that jaw. The load was applied by following a constant rate of extension. After a specific time, the fabric was broken apart. The final load was documented.

### Assessment of crease recovery properties

The unwanted or unintended fold mark on fabric at some stages of processing can be defined as a crease. It is a complex effect involving tensile, compressive, flexing, and torsional stresses. The fabric which has properties of crease recovery depends on its construction, twist of the yarn, pressure, time, etc. Here ASTM D1295 method was followed to determine the crease recovery angle. The sample size was kept at 40 mm X 15 mm (l X w) according to the standard. The sample was cut in both warp and weft directions using the relevant glass template. Then, the load was applied to the fabric for (5 ± 2) min to create crease. After that, the sample was clamped on the Shirley crease recovery tester for (5 ± 2) min. Finally, the crease recovery angle value was taken.

### Valuation of fabric weight

GSM stands for grams per square meter (g/m^2^). The GSM Cutter is a circular fabric sample cutter that is used as a textile testing tool for measuring fabric GSM using a weighing scale such as an electric balance and a GSM pad. The area of the fabric samples cut by the GSM cutter was 100 cm^2^. To convert it into gm/m^2^, the obtained value was multiplied by 100. Standard test method ASTM D 3776. (1996) was used to determine the mass per unit area (weight) of woven fabric for two samples.

### Statement of plant guidelines

The collection of *Eichhornia crassipes* (plant) material was performed according to institutional, national, and international guidelines. Plant studies and all experimental procedures were performed in conformity with applicable institutional, national, and international guidelines. Plants were identified by Md. Hashem, Associate Professor, Department of Botany, Khulna Govt. Girls College, National University, Bangladesh. (Herbarium number: 66769—*Eichhornia crassipes((Mart.)solms*.).

## Conclusion

The antimicrobial effect from the AgNPs-Chitosan coated cotton fabric where the AgNPs were derived from the e-waste through chemical and green synthesis way has been reported in this research work.

- The UV result clearly stated that nanoparticle formation occurred throughout both the chemical and green synthesis processes. It was found that the green synthesized AgNPs show higher absorbency of UV spectra compared to the chemically synthesized AgNPs due to plants' different phytochemical groups such as phenols, proteins, alkaloids, amino acids, carbohydrates, flavonoids, glycosides, tannins, and terpenoids.
- The FTIR results ensure that the AgNPs are perfectly bonded with the cellulose and chitosan. The results of FTIR analysis showed the presence of several foreign materials Cu, Zn, and Fe in recycled AgNPs coated fabric were confirmed from the bands at 678, 580, 457 cm^−1^. EDX analysis is also in line with this result.
- The SEM results showed spherical shape silver nanoparticles with a mean size of around 76.91 nm and a distribution range between 39.44 nm to 103.8 nm. SEM mapping also revealed a homogeneous distribution of Ag nanoparticles throughout the fabric. The particle size of AgNPs measured in XRD analysis confim the above finding of SEM analysis.
- The green synthesis yielded sound antimicrobial effects comparable to those of pure silver nanoparticles. However, the recycled AgNPs from e-waste did not exhibit good antimicrobial outcomes due to the larger particle size resulting from, the lower pH of the AgNO_3_ solution. Proper nanosized Ag particles with good antimicrobial results can be obtained by controlling the pH within the range of 7 to 12.
- Crease recovery angle of AgNPs-treated fabrics demonstrated significant changes compared to that of untreated fabrics. While other properties such as weight, thickness, and tensile strength increased at a minimum level.

Apart from this, the findings of this study demonstrate that green synthesis of AgNPs is promising and eco-friendly in comparison to chemical synthesis. By using recycled AgNPs derived from e-waste not only reduce the environmental impact but also contribute to sustainable manufacturing practices.

## Data Availability

All the data is embedded in the manuscript.
